# Community wide interventions for increasing physical activity

**DOI:** 10.1590/S1516-31802011000600013

**Published:** 2011-12-01

## Abstract

**BACKGROUND:**

Multi-strategic community wide interventions for physical activity are increasingly popular but their ability to achieve population level improvements is unknown.

**OBJECTIVES:**

To evaluate the effects of community-wide, multi-strategic interventions upon population levels of physical activity.

**SEARCH STRATEGY:**

We searched the Cochrane Public Health Group Specialized Register, The Cochrane Library, MEDLINE, MEDLINE in Process, EMBASE, CINAHL, LILACS, PsycINFO, ASSIA, The British Nursing Index, Chinese CNKI databases, EPPI Centre (DoPHER, TRoPHI), ERIC, HMIC, Sociological Abstracts, SPORT Discus, Transport Database and Web of Science (Science Citation Index, Social Sciences Citation Index, Conference Proceedings Citation Index). We also scanned websites of the EU Platformon Diet, Physical Activity and Health;Health-Evidence.ca; the International Union for Health Promotion and Education; the NIHR Coordinating Centre for Health Technology (NCCHTA) and NICE and SIGN guidelines. Reference lists of all relevant systematic reviews, guidelines and primary studies were followed up. We contacted experts in the field from the National Obesity Observatory Oxford, Oxford University; Queensland Health, Queensland University of Technology, the University of Central Queensland; the University of Tennessee and Washington University; and hand-searched six relevant journals. The searches were last updated to the end of November 2009 and were not restricted by language or publication status.

**SELECTION CRITERIA:**

Cluster randomized controlled trials, randomized controlled trials (RCT), quasi-experimental designs which used a control population for comparison, interrupted time-series (ITS) studies, and prospective controlled cohort studies (PCCS) were included. Only studies with a minimum six-month follow up from the start of the intervention to measurement of outcomes were included. Community-wide interventions had to comprise at least two broad strategies aimed at physical activity for the whole population. Studies which randomized individuals from the same community were excluded.

**DATA COLLECTION AND ANALYSIS:**

At least two review authors independently extracted the data and assessed the risk of bias of each included study. Non-English language papers were reviewed with the assistance of an epidemiologist interpreter. Each study was assessed for the setting, the number of included components and their intensity. Outcome measures were grouped according to whether they were dichotomous (physically active, physically active during leisure time and sedentary or physically inactive) or continuous (leisure time physical activity, walking, energy expenditure). For dichotomous measures we calculated the unadjusted and adjusted risk difference, and the unadjusted and adjusted relative risk. For continuous measures we calculated net percentage change from baseline, unadjusted and adjusted risk difference, and the unadjusted and adjusted relative risk.

**MAIN RESULTS:**

After the selection process had been completed 25 studies were included in the review. Of the included studies, 19 were set in high income countries, using the World Bank economic classification, and the remaining six were in low income countries. The interventions varied by the number of strategies included and their intensity. Almost all of the interventions included a component of building partnerships with local governments or non-governmental organizations (NGOs) (22 studies). None of the studies provided results by socio-economic disadvantage or other markers of equity consideration. However of those included studies undertaken in high income countries, 11 studies were described by the authors as being provided to deprived, disadvantaged, or low socio-economic communities. Fifteen studies were identified as having a high risk of bias, 10 studies were unclear, and no studies had a low risk of bias. Selection bias was a major concern with these studies, with only one study using randomization to allocate communities (Simon 2008). No studies were judged as being at low risk of selection bias although 16 studies were considered to have an unclear risk of bias. Eleven studies had a high risk of detection bias, 10 with an unclear risk and four with no risk. Assessment of detection bias included an assessment of the validity of the measurement tools and quality of outcome measures. The effects reported were inconsistent across the studies and the measures. Some of the better designed studies showed no improvement in measures of physical activity. Publication bias was evident.

**AUTHORS’ CONCLUSIONS:**

Although numerous studies have been undertaken, there is a noticeable inconsistency of the findings of the available studies and this is confounded by serious methodological issues within the included studies. The body of evidence in this review does not support the hypothesis that multi-component community wide interventions effectively increase population levels of physical activity. There is a clear need for well-designed intervention studies and such studies should focus on the quality of the measurement of physical activity, the frequency of measurement and the allocation to intervention and control communities.

Attending physician in the Discipline of Emergency Medicine and Evidence-Based Medicine, Universidade Federal de São Paulo, and Titular Professor of the Discipline of Rheumatology, Universidade de Santo Amaro, São Paulo, Brazil.

**This review was published in Cochrane Database of Systematic Reviews 2011, Issue 4. Art. No.:** CD008366. DOI: 10.1002/14651858.CD008366.pub2. For full details, see reference 1

**The full text of this review is available (open access) from:** http://www.cochranejournalclub.com/community-wide-interventions-for-increasing-physical-activity-clinical/pdf/CD008366.pdf.

**This section was edited under the responsibility of the Brazilian Cochrane Center – www.centrocochranedobrasil.org**

## COMMENTS

There is now a large amount of high-quality scientific evidence demonstrating the importance of physical activity for overall health. The benefits achieved have been demonstrated in various systems, such as the cardiovascular and osteoarticular systems. However, adherence and awareness among the population remain low in relation to the importance of physical activity as an adjunct to maintenance of health.

Multi-strategic community-wide interventions are of interest, since they can reach a large proportion of the population regardless of social class and socioeconomic level, and they are strategies that may help to spread knowledge about the benefits or harm of a habit or an intervention, thereby improving health. However, as was clearly demonstrated in this review, ^1^ which included a large number of studies, the results achieved failed to demonstrate the effectiveness of physical activity because of the lack of methodological rigor in the component studies. Nonetheless, lack of evidence does not in any way mean lack of effectiveness. On the other hand, unfortunately, this review generates a conclusion that cannot support the implementation of new interventions.

Because of the importance of physical activity, the result from this review should not be allowed to invalidate the possibility that other studies could be developed to answer this question better, with an adequate sample size corresponding to one population and appropriate ways of measuring the adherence to and benefits from physical activity; and with the commitment of a large enough number of researchers to be able to assist in conducting the trial.
